# High‐frequency electrical properties tomography at 9.4T as a novel contrast mechanism for brain tumors

**DOI:** 10.1002/mrm.28685

**Published:** 2021-02-02

**Authors:** Clémentine Lesbats, Nitish Katoch, Atul Singh Minhas, Arthur Taylor, Hyung Joong Kim, Eung Je Woo, Harish Poptani

**Affiliations:** ^1^ Centre for Preclinical Imaging Department of Molecular and Clinical Cancer Medicine University of Liverpool Liverpool UK; ^2^ Department of Biomedical Engineering Kyung Hee University Seoul South Korea; ^3^ School of Engineering Macquarie University Sydney NSW Australia

**Keywords:** conductivity, diffusion, DTI, EPT, MREPT

## Abstract

**Purpose:**

To establish high‐frequency magnetic resonance electrical properties tomography (MREPT) as a novel contrast mechanism for the assessment of glioblastomas using a rat brain tumor model.

**Methods:**

Six F98 intracranial tumor bearing rats were imaged longitudinally 8, 11 and 14 days after tumor cell inoculation. Conductivity and mean diffusivity maps were generated using MREPT and Diffusion Tensor Imaging. These maps were co‐registered with T_2_‐weighted images and volumes of interests (VOIs) were segmented from the normal brain, ventricles, edema, viable tumor, tumor rim, and tumor core regions. Longitudinal changes in conductivity and mean diffusivity (MD) values were compared in these regions. A correlation analysis was also performed between conductivity and mean diffusivity values.

**Results:**

The conductivity of ventricles, edematous area and tumor regions (tumor rim, viable tumor, tumor core) was significantly higher (*P* < .01) compared to the contralateral cortex. The conductivity of the tumor increased over time while MD from the tumor did not change. A marginal positive correlation was noted between conductivity and MD values for tumor rim and viable tumor, whereas this correlation was negative for the tumor core.

**Conclusion:**

We demonstrate a novel contrast mechanism based on ionic concentration and mobility, which may aid in providing complementary information to water diffusion in probing the microenvironment of brain tumors.

## Abstract 2

Click here for author‐reader discussions

## 1 INTRODUCTION

Glioblastomas (GBMs) are the most common type of malignant brain tumors, comprising approximately half of all the primary malignant tumors in the brain.^1^ Despite aggressive treatment including surgery followed by chemo‐radiation therapy, GBMs often recur leading to a dismal prognosis. While newer and more effective treatment strategies are being developed, the standard of care treatment is often sub‐optimal due to diffused margins and invasive nature of the tumor, which makes it difficult to assess the tumor margins for surgery or for radiation planning.

Magnetic resonance imaging (MRI) is widely used for diagnosis, treatment planning, and post‐surgical assessment of brain tumors.^1^, ^2^, ^3^, ^4^, ^5^, ^6^, ^7^, ^8^ Diagnostic MRI for characterization of brain tumors in human patients typically involves T_1_, T_2_, contrast enhanced T_1_ as well as diffusion‐weighted imaging (DWI). As per the current consensus for a standardized Brain Tumor Imaging Protocol (BTIP), the minimum recommended pulse sequences include: (1) pre‐contrast, axial 2‐dimensional (2D), T_2_‐weighted fluid‐attenuated inversion recovery (FLAIR); (2) pre‐contrast, 2D axial, 3‐directional (3D) diffusion‐weighted imaging (DW‐MRI); (3) parameter‐matched pre‐contrast and post‐contrast inversion recovery‐prepared, isotropic 3D T_1_‐weighted gradient‐recalled echo (DCE‐MRI); and (4) 2D axial, T_2_‐weighted turbo spin‐echo acquired after contrast injection and before post‐contrast 3D T_1_‐weighted images.^2^ While the BTIP is helpful in the diagnosis, difficulties arise in case of non‐enhancing GBMs^4^ or in monitoring treatment response, especially in differentiating pseudo‐progression from tumor recurrence.^5^

A better understanding of GBM heterogeneity and invasion using imaging methods will not only aid in diagnosis, but also help in designing targeted treatment strategies. Over the recent years, several MR methods have been proposed to assess the GBM microenvironment including sodium imaging for alterations in sodium^3^ and ^1^H magnetic resonance spectroscopy (MRS) for tumor metabolism,^6^ spin‐lattice relaxation in the rotating frame (T1ρ) for biological heterogeneity,^7^ and chemical exchange saturation transfer (CEST) MRI to measure the pH^8^ and protein content of tumors.^8^ However, the quest for newer and better contrast mechanisms continue to provide additional or complimentary information about the tumor microenvironment.

There is a growing interest to exploit MR‐based electrical conductivity imaging to better understand tumor physiology.^9^, ^10^ This is a novel contrast mechanism reflecting changes in the ionic content of tissues.^11^, ^12^ Current injection‐based techniques were initially proposed to image electrical conductivity at low frequencies.^13^ Recently, Magnetic Resonance Electrical Properties Tomography (MREPT) has been developed to measure tissue conductivity and permittivity at the resonance frequency of MRI scanner without the need of any external electrical current.^11^, ^12^ Several algorithms have been proposed for MREPT image reconstruction,^14^, ^15^, ^16^ including the most recent machine learning‐based algorithms,^17^, ^18^ with an assumption that conductivity and permittivity are isotropic and piecewise constant within the imaging field of view (FOV). Of these, the phase‐based MREPT is the most popular method for MREPT image reconstruction.^16^, ^19^, ^20^

The first *in vivo* MREPT study of normal adult human brain was reported by Voigt et al at 1.5T.^16^ Subsequent MREPT studies reported conductivity values of normal human brain tissues such as white matter (WM), grey matter (GM) and cerebrospinal fluid (CSF) at 1.5T,^21^ 3T^21^ and 7T.^21^, ^22^, ^23^ Tha et al reported *in vivo* conductivity of grade II, grade III and grade IV gliomas at 3T.^9^ In that study, they validated MREPT‐based conductivity values by correlating them with probe‐based conductivity measurements and reported a moderate positive correlation between the mean MREPT conductivity of the resected tumor and the probe‐based conductivity measurements. Negative correlation was reported at 3T between apparent diffusion coefficient (ADC) and conductivity in rat models of breast and prostate carcinomas^10^ and in invasive ductal carcinoma.^24^ Kim et al reported an association between conductivity and overexpression of HER‐2 (human epidermal growth factor receptor 2) in breast cancer studies.^25^

The first preclinical MREPT study of subcutaneously implanted AT‐1 rat prostate cancer grown on the hind limb and studied at 7T reported a detectable increase of conductivity inside the tumor tissue compared with the nearby healthy muscle, which was consistent with the direct measurements of tumor and muscle conductivity using a dielectric probe used immediately following euthanasia.^26^ A recent study investigated the feasibility and accuracy of MREPT‐based conductivity and permittivity measurements at 21.1T in phantoms of different sodium chloride (NaCl) concentrations.^27^ This study was performed using linear birdcage and quadrature saddle coils comparing the Helmholtz‐based full‐form reconstruction with the phase‐based conductivity reconstruction.^27^ While the reported errors were high, they were in agreement with the previous reported errors by Katscher et al.^14^ Additionally, the in‐vivo rat brain conductivities of ischemic stroke were higher than the naïve conditions, and this result was independent of the two reconstruction algorithms, showing no difference between the phase‐based conductivity reconstruction and the Helmholtz‐based full‐form reconstruction.^27^

Previous studies have primarily reported MREPT‐based conductivity images using wide‐bore human MRI scanners from 1.5T to 7T and pre‐clinical scanners of 7T and 21.1T. However, no *in vivo* MREPT studies of brain cancer have been reported using 9.4T MRI. Imaging at ultra‐high fields (≥7T) with higher spatial resolution can provide a better visualization of tumor heterogeneity than lower fields, and permit increased dynamic range for the conductivity measurements.^10^ In this work, we report the electrical conductivity of a rat model of GBM at 9.4T. To our knowledge, no other study has investigated changes in electrical conductivity in rat models of gliomas, as they grow over time, to potentially evaluate changes in conductivity as the tumor tissue heterogeneity varies. The conductivity results were compared with diffusion tensor imaging (DTI) based mean diffusivity (MD) values.

## 2 METHODS

### 2.1 Animal model

*In vivo* studies on rats were conducted in compliance with the UK Home Office Animals (Scientific Procedures) Act 1986 and with the ethical approval of the local animal welfare committee. Intracranial tumors were induced by transcranial injection of F98 cells (ATCC CRL‐2937^TM^) in the right cortex. Six Fischer 344 female (100‐120 g) rats (Charles River, Margate, United Kingdom) were injected with 50,000 F98 cells in the cortical region.^28^ The injection was performed in an aseptic environment using sterile tools. The rat was maintained in a three‐point stereotaxic frame under surgical anesthesia using 3% isoflurane in O_2_ gas mixture. The head was shaved, and a small incision allowed access to the skull. A burr hole was drilled through the skull 3 mm right and 3 mm posterior from the bregma and the cells were injected 2.5 mm deep into the cerebral cortex. After the surgery, the skin was sutured, and the animal was returned to its cage for recovery.

### 2.2 MRI data acquisition

Longitudinal MRI was performed on 8, 11 and 14 days after implanting tumor cells using a 9.4T Biospec MRI scanner (Bruker Biospin, Inc. Germany). An 86 mm transmission birdcage coil was used to transmit RF signal and a 4‐channel phased array surface coil was used for receiving signal. Multi‐slice T_2_‐weighted images in the coronal plane were acquired to locate the tumor regions in rat brain. For MREPT, we acquired 38 slices (slice gap = 0) covering the tumor regions using a multi‐spin‐multi‐echo (MSME) pulse sequence (Figure S1, supporting information section A1) with following parameters: TR = 4341 ms, first TE = 8 ms, echo‐spacing = 8 ms, 10 echoes, 2 averages, FOV = 40 × 20 mm^2^, matrix = 128 × 64, number of phase encoding steps = 64, slice thickness = 0.3 mm, resolution = 0.313 × 0.313 × 0.3 mm^3^, scan duration = 9 min 15s. No cardiac gating was performed to account for pulsation effects. For comparative purposes, we used MD values from an *in vivo* DTI sequence which was acquired in the same imaging session. The DTI study was performed using single‐shot EPI pulse sequence with parameters: TR/TE = 2500/23 ms, 5 averages, b‐value = 0, 1000 and 2000 s/mm^2^, 15 directions, and respiratory triggered data acquisition with scan duration of 55 minutes. For *ex vivo* imaging, high‐resolution T_2_‐weighted, DTI and MREPT were conducted with a cubic voxel of 0.2 mm edge length (resolution = 0.2 × 0.2 × 0.2 mm^3^) in coronal plane.

### 2.3 Data processing

The mean diffusivity (MD = $\frac{\lambda_{1}+\lambda_{2}+\lambda_{3}}{3}$) maps were calculated from the DTI data. High‐frequency conductivity ($\sigma_{H}$) maps were reconstructed from the measured B1 phase maps acquired using the MSME sequence.^16^, ^19^, ^29^ The phase data from the different receive coils were combined using a two‐step generic referenceless phase combination (GRPC) method,^30^, ^31^, ^32^ described in detail in supporting information section A2. Prior to the conductivity image reconstruction, the acquired B1 phase map was unwrapped using PUMA algorithm.^33^ The multi‐echo data were then averaged using a weight‐factor in order to reduce noise,^19^ the weighting scheme is described in equation A4 and A5 in supporting information (section A2). The optimized B1 phase map was then used to reconstruct the high‐frequency conductivity by numerically solving Equation (1).^29^

(1) $$‐c\nabla^{2}\left(\frac{1}{\sigma_{H}}\right)+\nabla \phi_{\mathrm{tr}}\cdot \nabla \left(\frac{1}{\sigma_{H}}\right)+\nabla^{2}\phi_{\mathrm{tr}}\left(\frac{1}{\sigma_{H}}\right)=2\mu_{0}\omega$$

where, c is a constant diffusion coefficient to suppress oscillations in reconstructed image $\sigma_{H}$, and $\phi_{\mathrm{tr}}$ is transceive B1 phase map. Mean diffusivity and conductivity maps were co‐registered with T2‐weighted (b0) images and used to perform statistical analysis. These b0 images were acquired as part of the DTI data set without using the diffusion sensitizing gradient.

### 2.4 Tissue segmentation and statistical analysis

**FIGURE 1:**
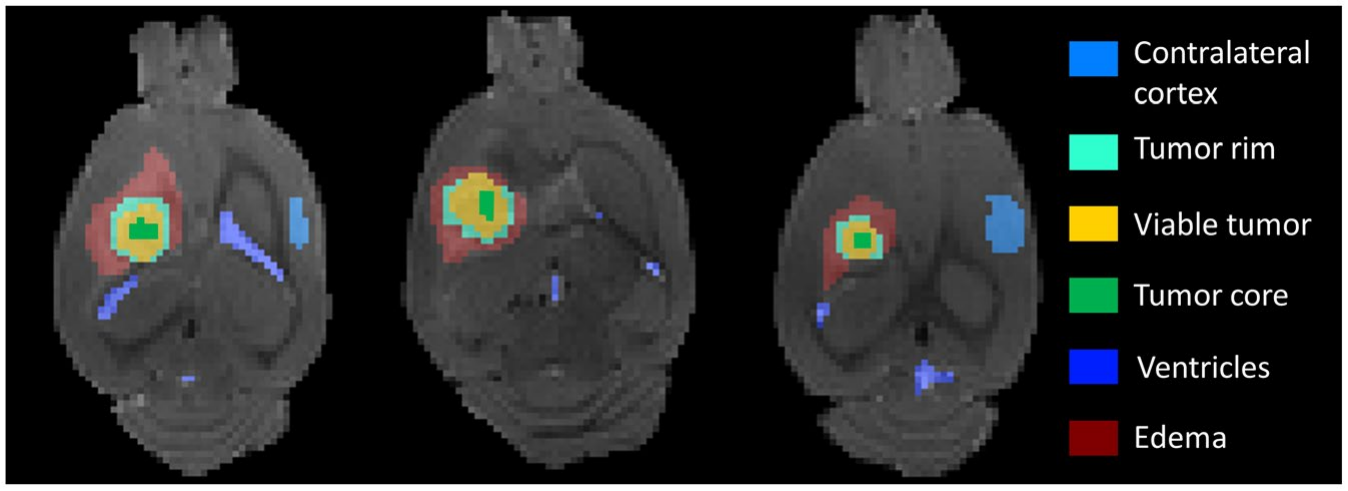
Segmented ROIs overlaid on T_2_‐weighted (b0) images for 3 representative rats. The color labels corresponding to six different segmented regions are shown on the right side of the images

Manual segmentation was performed from different slices to generate six volume of interests (VOIs) including the tumor core reflecting necrotic center, tumor rim reflecting the invasive areas of tumor, viable tumor reflecting the area between rim and core, which comprised of the major tumor mass, peritumoral edema, ventricles and contralateral normal brain cortex. The masks for these regions were manually segmented from the b0 images of the DTI data set for each slice encompassing these regions for each animal. The specific number of slices encompassing tumor for each rat and time point are shown in Table 1. Representative ROIs are depicted in Figure 1 on a slice with the maximum size of tumor for three representative rats. Using these masks, the average value of high‐frequency conductivity and MD was calculated for each animal in these regions for each time‐point in the longitudinal scans.

**TABLE 1:** Number of slices used to calculate tumor VOIs for each of the six rats and for each time point (Day‐8, Day‐11, Day‐14)

	Rat #1	Rat #2	Rat #3	Rat #4	Rat #5	Rat #6
Day‐8	9	11	12	14	11	7
Day‐11	10	13	14	16	13	7
Day‐14	15	18	16	19	16	11

The differences between conductivity and MD values in contralateral cortex, the segmented tumor regions (tumor core, viable tumor, and tumor rim), ventricles, and peritumoral edema on a given day were compared using two‐tail t‐tests. Variation of conductivity and MD in these regions across different time‐points was also evaluated by performing two‐tailed t‐tests between the distributions of conductivity at any two time‐points. Pearson’s correlation was performed between MD and conductivity by grouping the diffusivity and conductivity values of all the segmented regions at all the time‐points.

## 3 RESULTS

**FIGURE 2:**
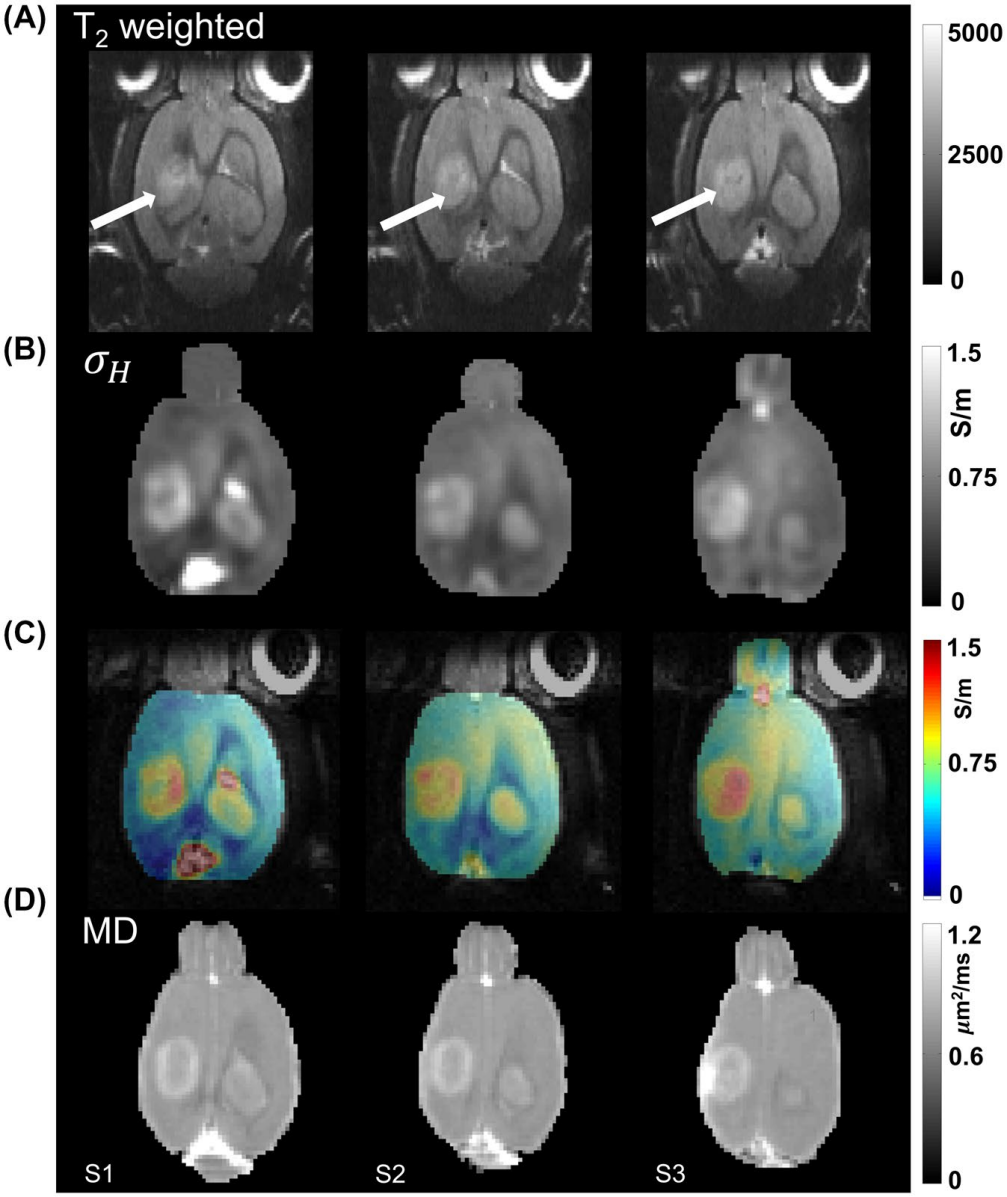
Three slices (S1‐S3) of the brain covering the widest extent of tumor volume are shown for day 8 of a representative rat. The images in (A) are T_2_‐weighted MR images, (B) shows the high frequency conductivity images from MREPT, (C) shows the high frequency conductivity overlaid on magnitude images, and (D) shows the mean diffusivity images. Arrows in (A) indicate the location of the tumor

**FIGURE 3:**
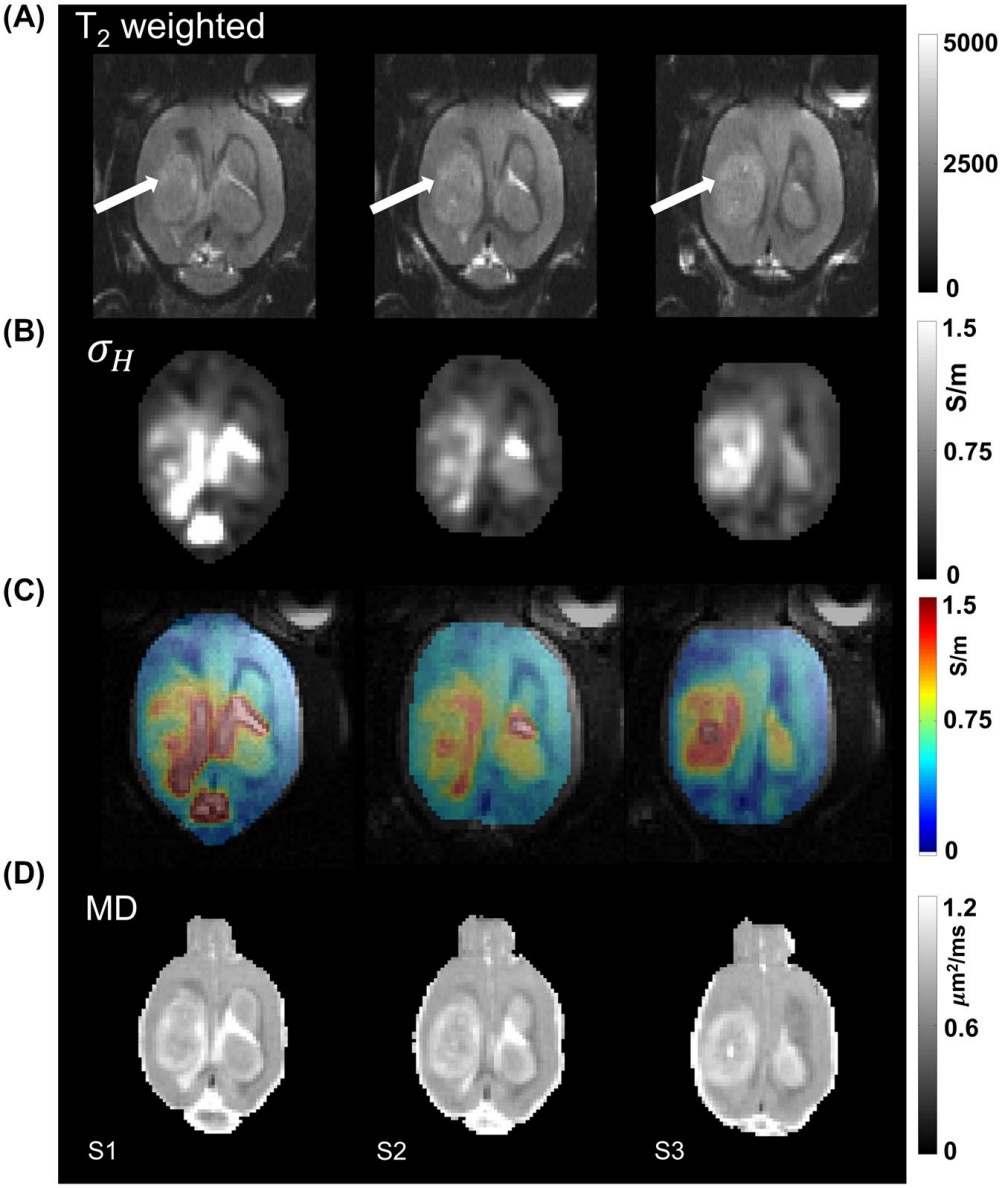
Three slices (S1‐S3) of brain covering the widest extent of tumor volume are shown for day 11 of the same rat as in Figure 2. The images in (A) are T_2_‐weighted MR images, (B) shows the high‐frequency conductivity images from MREPT, (C) high frequency conductivity overlaid on magnitude images, and (D) shows the mean diffusivity images. Arrows in (A) indicate the location of the tumor

Figure 2 shows *in vivo* images of a representative rat (Rat 1) 8 days after tumor cell injection. Only three slices (S1‐S3), which covered the widest extent of the tumor are shown. The images in (A) are T_2_‐weighted images where location of the tumor is pointed by arrows. The conductivity images in (B) are co‐registered with the averaged magnitude images of MSME and shown as color overlays in (C) for better visualization of the tumor. The corresponding MD images are shown in (D). The tumor rim and edema in conductivity images S1‐S3 of Figure 2B,C show higher conductivity and MD values than the central necrotic region of the tumor and the contralateral cortex region. Figure 3 shows *in vivo* image slices (S1‐S3) of the same representative rat (Rat 1) as the one shown in Figure 2, but on the 11^th^ day after tumor cell injection. The image positions S1‐S3 in Figure 3 were chosen similar to the positions S1‐S3 in Figure 2. The conductivity values were much higher in ventricles than in other tissues, as can be noticed from the day 11 images in Figure 3B. Similar to Figure 2, color overlays are shown in Figure 3C, while comparative MD images are shown in Figure 3D. The ventricles appear enlarged in Figure 3 due to excessive fluid accumulation due to restriction in CSF flow (hydrocephalus) caused by the tumor.

**FIGURE 4:**
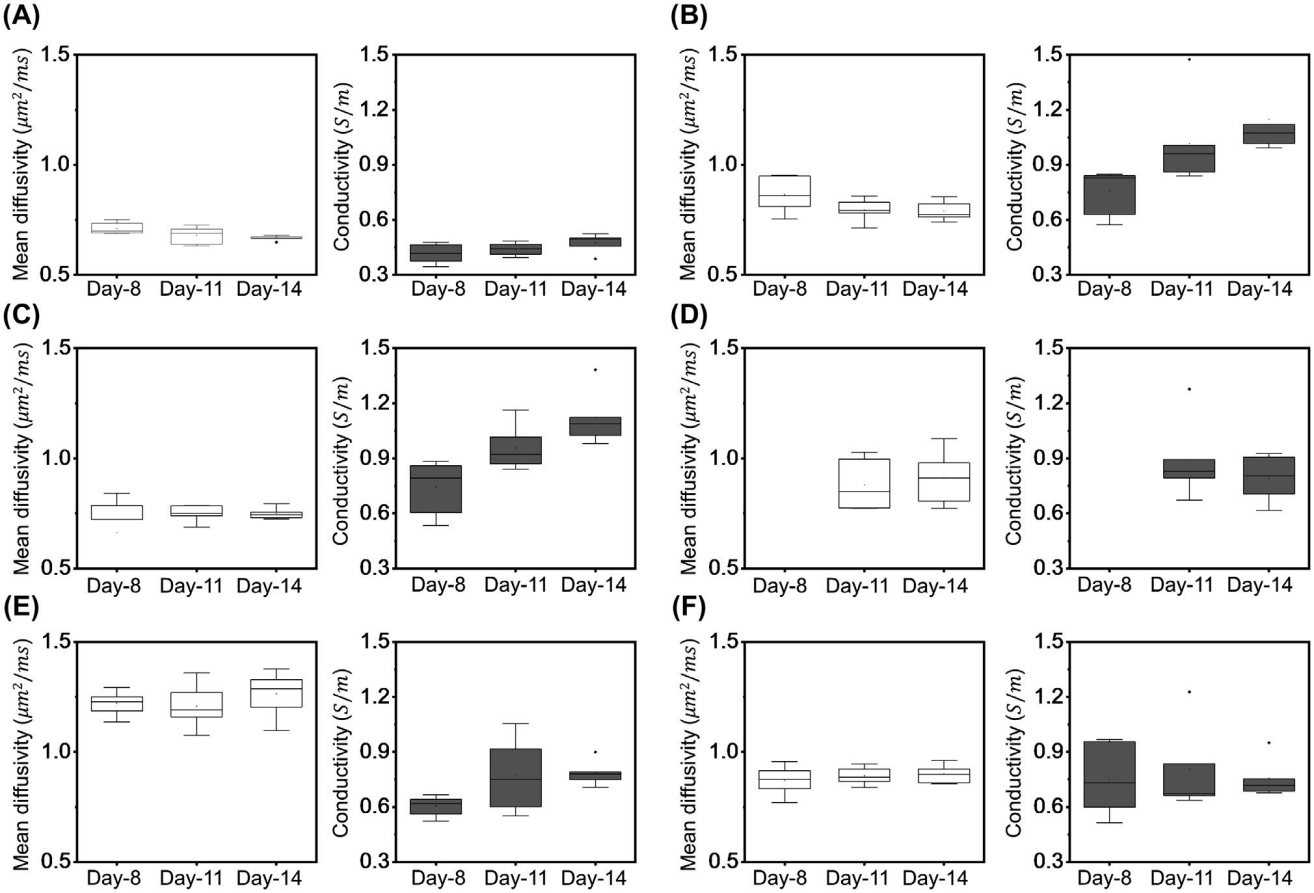
Boxplots displaying MD and conductivity of six regions segmented from six rats. The regions of interest for these six segmented regions are illustrated in Figure 5 (A). Each boxplot from (A)‐(F) shows measurements on day 8, day 11, and day 14 for contralateral cortex, tumor rim, viable tumor, tumor core, ventricle, and edema, respectively. The tumor core was not visible on day 8 in all rats, and hence, these values are missing in (D). Vertical bars indicate the range of data except outliers, rhombus marks outliers, box marks the 25^th^‐75^th^ percentile, whisker marks the standard deviation and horizontal bar marks the median

**FIGURE 5:**
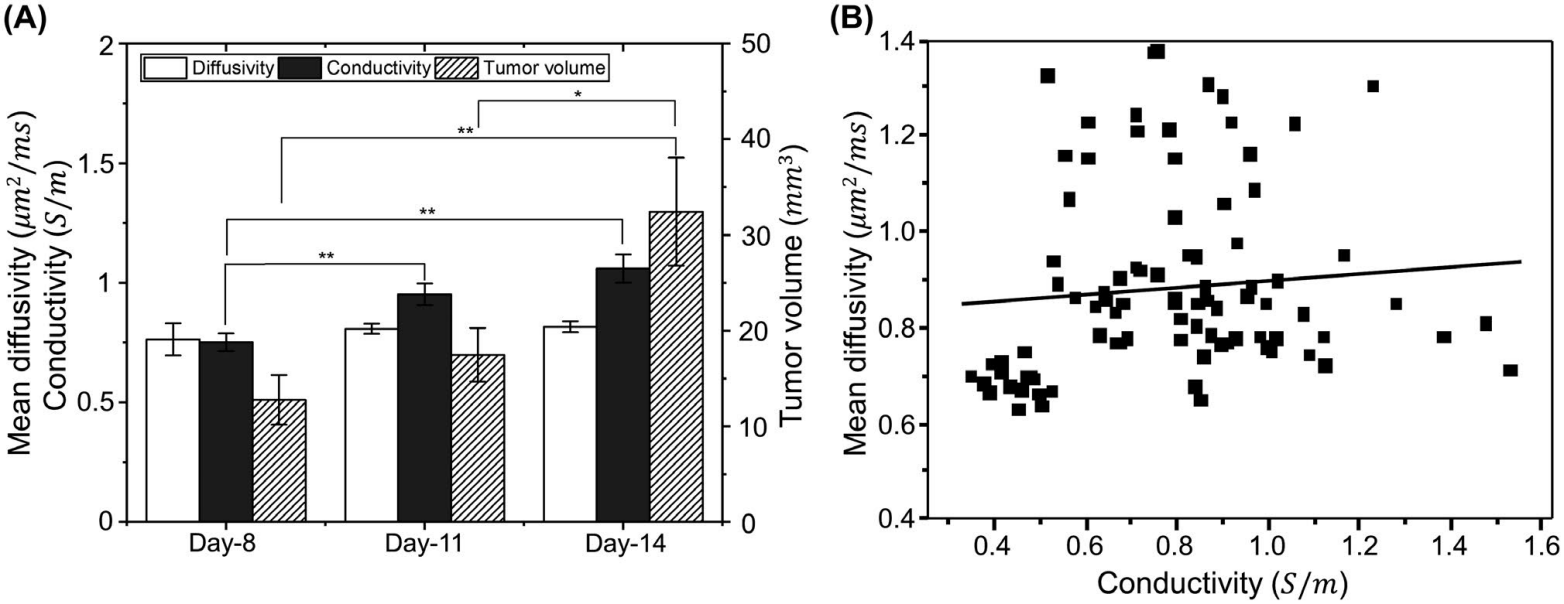
Longitudinal data in (A) shows the variation of MD, conductivity and tumor volume from day 8 to day 14. Error bars represents the standard error. The plot in (B) shows correlation between MD and conductivity for a combined data taken over all the time‐points and all the rats

**FIGURE 6:**
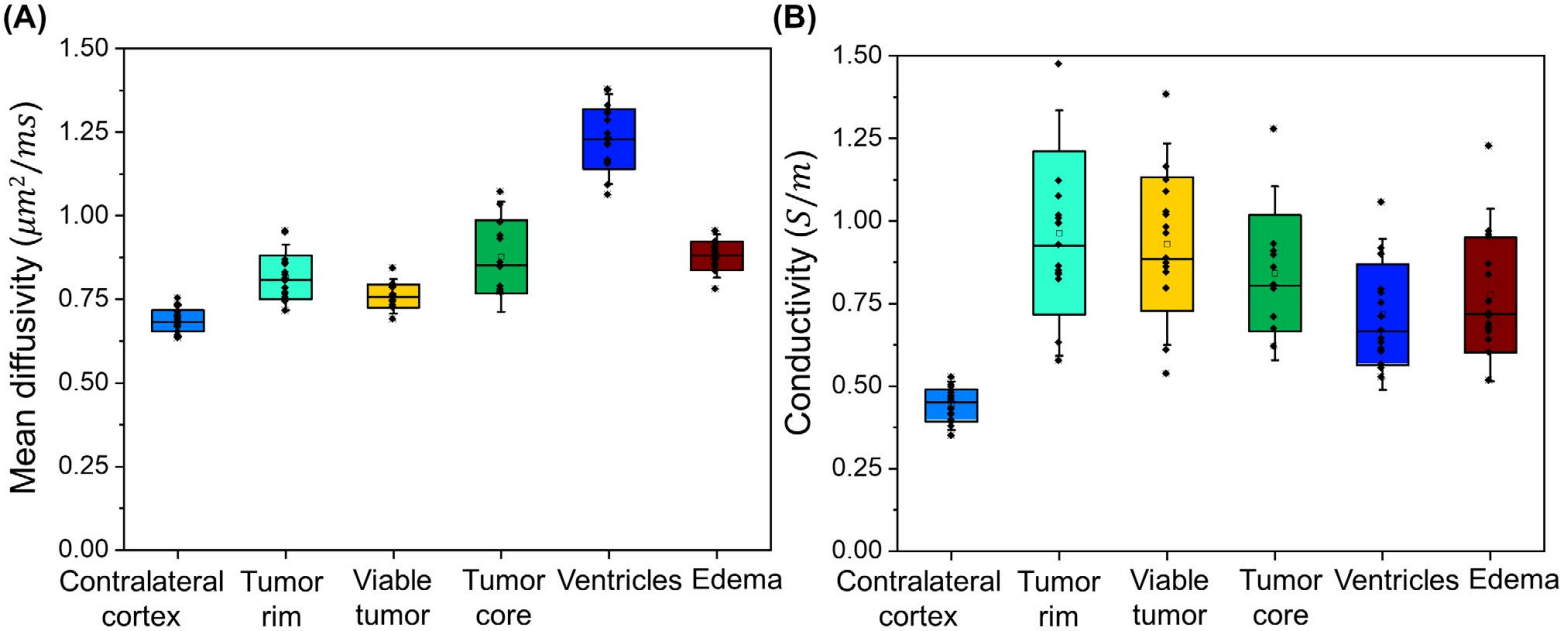
Comparison of the mean diffusivity and conductivity values in each of the six segmented tissue volumes of interests by combining data from all the time‐points. Boxplots of mean diffusivity are shown in (A), and boxplots of conductivity are shown in (B). The color in box plot (A) and (B) corresponds to the color of segmented tissues shown in Figure 1. Vertical bars mark the range of data excepting outliers, rhombus marks the measured mean values, starred‐rhombus marks outliers, box marks the standard deviation, whisker marks the standard deviation with coefficient = 1.5, and horizontal bar marks the median

Results of statistical analysis for the six animals are shown in Figures 4, 5, 6 for six different regions: contralateral normal brain cortex, tumor rim, viable tumor, tumor core, ventricles, and edema. Figure 4 shows the box plots of the conductivity and MD values from the six segmented regions across 8^th^, 11^th^ and 14^th^ day after tumor cell injection. As the necrotic core was not evident in all rats on day 8, Figure 4D does not show the conductivity and MD values for this day. The conductivity of ventricles, edematous area and tumor regions (tumor rim, viable tumor, tumor core) was significantly higher (*P* < .01) compared to the contralateral cortex on each of the 8^th^, 11^th^ and 14^th^ day after injecting tumor cells. The MD values from the ventricles and edematous tissues were also significantly higher (*P* < .01) than contralateral tissues for all the three days. Although there was no significant change (*P* = .59) in the whole tumor (tumor rim, viable tumor, tumor core) MD values compared to the contralateral cortex on day 8, they became significantly higher (*P* < .01) on the 11^th^ and 14^th^ day. Comparing the conductivity of edematous region and tumor regions, no significantly different conductivity was observed (*P* > .17) on the 8^th^ and 11^th^ day, but the conductivity of tumor increased significantly on day 14^th^ (*P* < .01). The MD values of edema were similar to tumor rim and viable tumor on day 8 (*P* > .12) but increased significantly on day 11 and day 14 (*P* < .01).

Focusing only on the tumor regions (tumor rim, viable tumor, tumor core) in Figure 4, we found that their MD values did not change significantly (*P* > .39) from day 8 to day 14, but there was a significant increase (*P* < .02) in the corresponding conductivity values. This same trend can also be visualized in Figure 5A for the average values of MD and conductivity in tumor regions. We also found that the tumor volume did not change much from day 8 to day 11 (*P* = .24) but increased significantly (*P* < .04) from day 11 to day 14. We observed a marginal positive correlation between MD and conductivity when we combined the data from all the time‐points for the six segmented regions with a Pearson’s correlation coefficient of r = 0.09, as can be seen in Figure 5B.

Since there was no significant change over time in the MD values of tumor regions we combined data from all time‐points for each of the six segmented regions to analyze the difference in conductivity and MD values regardless of tumor size (Figure 6A,B). Similar to the results of uncombined data, the conductivity of ventricles, edematous tissues and tumor regions in the combined data was significantly higher (*P* < .01, Table 2) than the contralateral cortex. Likewise, the MD values were also significantly higher (*P* < .01) for ventricles, edematous tissues and tumor regions than the contralateral cortex in the combined data (Table 2). The tumor rim conductivity value was significantly higher than the ventricles (*P* < .01) and edema (*P* < .02) but was similar (*P* > .17) to the other regions (viable tumor and tumor core). The tumor rim MD was significantly lower than ventricles (*P* < .01), edema (*P* < .01), and tumor core (*P* < .03), but significantly higher than viable tumor (*P* < .04). The MD of ventricles was also significantly higher (*P* < .01) than edema, but their conductivity values were similar (*P* < .37).

**TABLE 2:** Average values of MD and conductivity after combining VOIs for all the time‐points

	Contralateral cortex	Tumor rim	Viable tumor	Tumor core	Ventricles	Edema
Conductivity (S/m)	0.44 ± 0.05	0.96 ± 0.25	0.93 ± 0.20	0.84 ± 0.17	0.72 ± 0.15	0.77 ± 0.18
Mean Diffusivity (μm^2^/ms)	0.69 ± 0.03	0.81 ± 0.06	0.76 ± 0.03	0.89 ± 0.11	1.23 ± 0.08	0.89 ± 0.05

**FIGURE 7:**
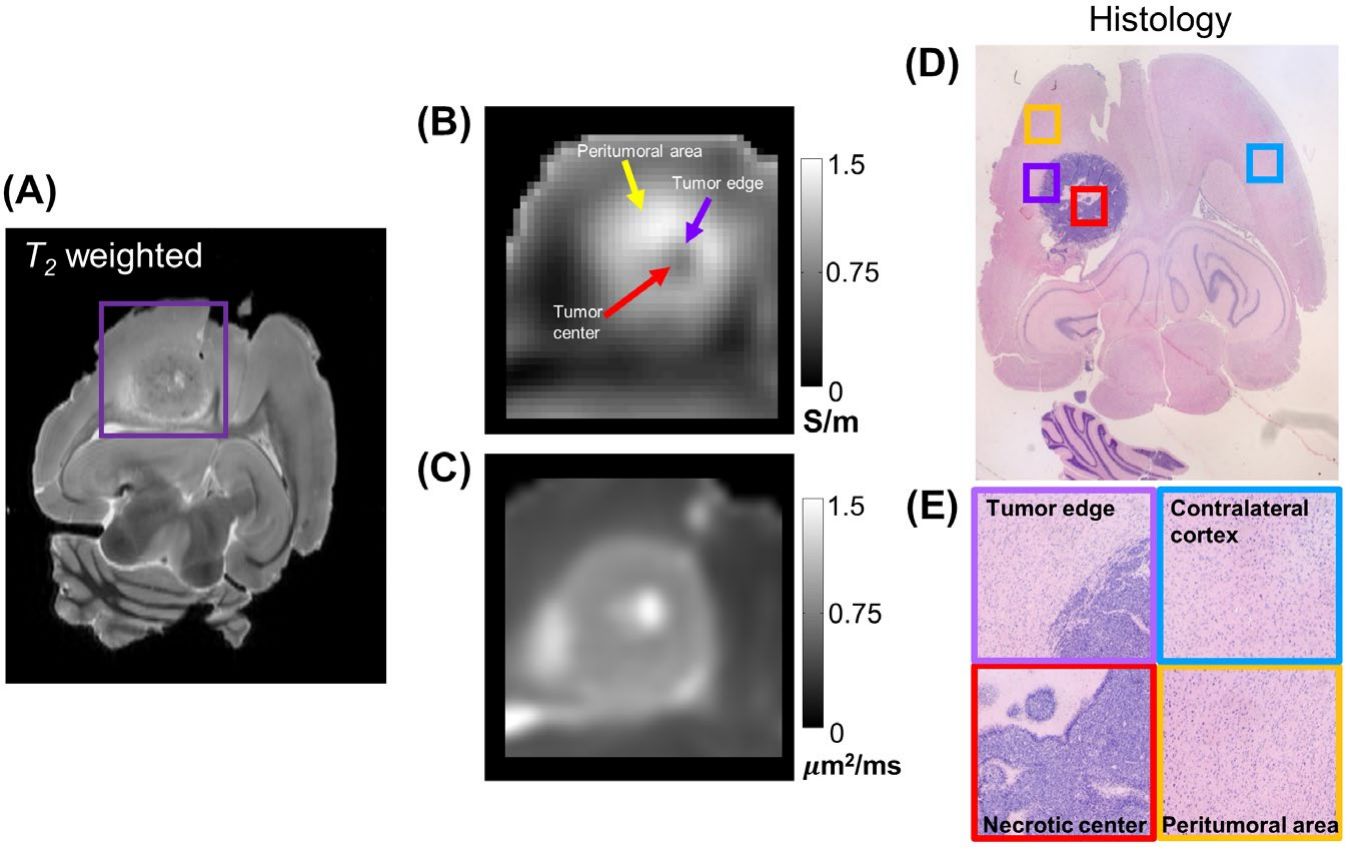
*Ex vivo* MR imaging and H&E images of rat brain. (A) shows a T_2_‐weighted MR image from the similar region as the histological image in (D), (B) and (C) are high frequency conductivity and MD images corresponding to the region marked by a purple color box in (A) with a matrix size of 64 × 64. (D) shows H&E stain with colored boxes representing the magnified regions in (E)

We also conducted high‐resolution *ex vivo* MRI of the rat brain of one animal using the same sequence parameters but at higher resolution and increased number of averages followed by histological analysis using H&E staining. Figure 7A shows a T_2_‐weighted image of 0.2 × 0.2 mm^2^ resolution from the *ex vivo* data. The regions: tumor core, tumor edge, and peritumoral area are labeled in (B), which is a magnified version of the conductivity image corresponding to the purple colored box in (A) and extended with a resolution of 0.2 × 0.2 mm^2^. The corresponding region for MD is shown in (C). The H&E stain image corresponding to the T_2_‐weighted image is shown in (D). Small squares are marked on (D) with different colors and their magnified view is shown in (E). We can notice clear boundaries between healthy tissue and tumor edge, and between tumor and the necrotic tumor core.

## 4 DISCUSSION

We demonstrated that MREPT‐based high‐frequency conductivity can be used as an additional imaging contrast for tumor characterization. Longitudinal changes in conductivity were compared with changes in MD since mean diffusivity (or ADC) has been studied extensively preclinically.^34^, ^35^, ^36^ While no correlation was noted between high frequency conductivity and MD, the conductivity values from the whole tumor increased linearly as the tumor grew during which time MD values remained unchanged. These results suggest that conductivity values provide complementary information about the tumor microenvironment.

Electrical conductivity is primarily determined by the product of the ion concentration and mobility. While the effect of the ionic concentration is independent of frequency, the effect of mobility depends on the frequency especially for those ions in the extracellular fluid since their movements at low frequency are hindered by the cell membranes. This frequency dependence in ionic mobility is an origin of anisotropy at low frequency. Considering that ions are surrounded by numerous molecules, their mobility should change with frequency in general. Since we used a fixed high frequency of 400 MHz, the conductivity contrast reported in this study is mainly due to differences in the ion concentrations of different tissues.

The conductivity values of tumor core and tumor rim observed in our study are within the range of the previously reported values by Tha et al for grade IV tumors in the human brain.^9^ The higher conductivity in the tumor regions (tumor rim, viable tumor and tumor core) in our study, in comparison to the contralateral cortex, suggests increased ionic concentration in the tumor compared to the normal brain. Interestingly ^23^Na‐MRI of brain tumors also report increased contrast indicating an increased Na+ concentration and imbalance in the Na+/K+ pumps in brain tumors.^37^, ^38^, ^39^ Since the high frequency conductivity reflects a change in ionic concentration, it is possible that higher conductivity in tumor regions may reflect increased sodium concentration. Higher conductivity in the ventricles and edema compared to the contralateral cortex could be due to increased ionic mobility from the higher fluid content in these areas which was confirmed by their high MD values.

When data from all the time‐points were combined (Figure 6), significantly lower conductivity was observed in edematous region than the tumor rim, while the MD was high. This suggests that the water molecules were more mobile in edema but had lower ionic concentration than in tumor rim, which had a higher concentration of freely movable ions contributing to higher conductivity, which typically reflects the contrast‐enhancing invasive regions of the tumor. Although preliminary, these results suggest that conductivity measurements can provide complementary information in differentiating tumor from edema, especially in non‐enhancing tumors, where it is difficult to estimate the tumor margins.^4^

Higher MD values in tumor compared to the contralateral cortex reflect increased extracellular volume fraction. Wang et al reported the mean diffusivity values of tumor core as 0.87 ± 0.04 and tumor rim as 0.87 ± 0.05 µm^2^/ms in their F98 rat tumor models^36^ which are very similar to our data of 0.81 ± 0.06 µm^2^/ms for tumor rim and 0.89 ± 0.11 µm^2^/ms for tumor core.

There was no significant change from day 8 to day 11 in the MD values of tumor regions (tumor rim, tumor core) but the conductivity values increased significantly. This is anticipated for MD values since it reflects extracellular volume fraction and is less sensitive for assessment and delineation of tumor cell infiltration.^40^ Our observation of increased conductivity in tumor while stable MD values is consistent with reports from Schepkin et al,^34^ where a steady increase in average Na+ concentration of the tumor was reported without any significant change in ADC values despite the steady growth in tumor volume. The significant increase in the conductivity observed longitudinally indicates that there was a significant increase in the number of tumor cells as tumor grew in size, which could imply an increase in the Na+ ion concentration from day 8 to day 14.^37^, ^38^, ^39^ This indicates that a potential correlation exists between conductivity and Na+ concentration in tumor regions,^41^, ^42^ but further investigation with a correlative study between MREPT and Na+ imaging is needed to fully establish its basis. The contrast in the longitudinal values of conductivity suggests that conductivity imaging has the potential to distinguish between the different grades of tumors where size and tumor microenvironment changes over time.

We found no correlation between the mean diffusivity and conductivity when all regions were evaluated, but a positive correlation was noted when only the tumor regions were analysed. This result was similar to the low‐frequency conductivity studies by Haueisen et al,^43^ but in contrast to the negative correlation found by Hancu et al^10^ in breast and prostate carcinomas induced in rats. This discrepancy could be due to the difference in the tissues chosen and the conductivity measurement method. Hancu et al calculated the correlation between probe‐based conductivity measurements in the necrotic regions of the tumor and the corresponding ADC values from MRI. We can notice from Figure 6A,B that MD of tumor core is higher than tumor rim and viable tumor, but the conductivity of tumor core is lower, which indicates that a negative correlation existed between MD and conductivity within tumor core or necrotic regions in our results as well.^44^, ^45^ The necrotic region in our *ex vivo* data did in fact demonstrate a negative correlation between MD and conductivity values (data not shown). It has been reported in other studies that MD increases in necrotic tissue due to reduced cell density and ruptured cell membrane but its conductivity decreases due to low ionic concentration.^46^, ^47^ It should also be noted that we calculated correlation between MD and conductivity for all VOIs, whereas Hancu et al did probe‐based measurements only on carcinomas which are at the periphery of rat body. Therefore, our results provide a more generic relationship based on water diffusion and ionic concentration with a broader range of conductivities of tissues used. Additionally, we also observed that the conductivity contrast was much higher in the ventricles, which combined with the larger range of conductivity values, may be highly sensitive for detecting hydrocephalus. Conductivity values typically range between 0.6 S/m and 0.9 S/m at 128 MHz (3T),^10^ the commonly used clinical field strengths. However, since conductivity increases with frequency, one may get higher dynamic ranges of 0.3 S/m to 1.6 S/m at 400 MHz (9.4T), the field strength used in the current study.^48^, ^49^, ^50^ The increased dynamic range of conductivity values potentially makes the technique more sensitive in detecting smaller changes in pathology or for detecting treatment response to novel therapeutics.

Although promising, using MREPT to characterize brain tumors has its limitations due to its dependency on accurate measurement of the phase information from each image. While not a major issue at low magnetic fields, it is difficult at high and ultra‐high magnetic fields due to increased B1 inhomogeneity. The phase‐based MREPT image reconstruction algorithm, used in this work, relies on Laplacian calculation, which creates blurring at the boundary between two distinct tissues. This is not the case with DTI which provides clear distinction between tissue boundaries. However, measured MD values could be influenced by the choice of b‐value in experimental conditions. The phase‐based MREPT image reconstruction required a coil‐combination algorithm to create one phase image from a combination of 4‐channel data for each slice and each echo. While we used the algorithm from previously published studies,^30^, ^31^ we do not think that the results of this work would change significantly if other algorithms are used for coil‐combination. Nevertheless, comparison of the effects of various coil‐combination algorithms is worth an investigation in future.

Progress has been made over the past decade and MREPT has improved since the early studies reported at the beginning of this decade.^14^, ^15^, ^16^, ^51^ With the trend of using ultra‐high magnetic fields of 7T for clinical use, increased sensitivity due to larger dynamic range of conductivity values through the MREPT method could find its acceptance in clinical studies for tumor characterization, especially when ion concentration is of interest in addition to water diffusion. Nevertheless, we believe that measurement of ionic conductivity from a relatively fast and simple multi‐echo T_2_‐weighted sequence, as used in the current study, provides a novel contrast for characterization of brain tumors and that this information may be complementary to other contrast mechanisms including mean diffusivity and may aid in better understanding of the biology of brain tumors.

In conclusion, conductivity imaging can add more valuable diagnostic information in non‐invasive preclinical and clinical practice. Findings of this study shows the potential of conductivity imaging technique as a useful biomarker for glioblastoma models. In future studies, combination of sodium and ^1^H MREPT‐based conductivity imaging could be helpful in identifying the underlying ionic mechanism dominant for conductivity contrast.

## Supporting information


**FIGURE S1** Multi‐spin‐multi‐echo (MSME) pulse sequence diagram for coronal imaging plane
**FIGURE S2** MREPT of phantom at 9.4T MRI. (A) Magnitude image illustrating the two electrolytes #1 and #2, and background. (B) B_1_ phase map after applying echo‐combination algorithm, (C) the reconstructed conductivity image. The (D) shows the profiles of B_1_ phase (left) and magnitude (right), along the red and black lines marked on cross‐sectional regions at the top‐right corner of the four graphs
**FIGURE S3** Conductivity spectra of the two electrolytes and the background agarose gel used in our phantom experiments from 10 Hz to 3 MHz. The conductivity values form the MREPT experiment also displayed at 400 MHz
