# Supplementary material for: High‐frequency electrical properties tomography at 9.4T as a novel contrast mechanism for brain tumors
Source: Magn Reson Med. 2021 Feb 2;86(1):382–92. doi: 10.1002/mrm.28685 (PMC8603929; doi:10.1002/mrm.28685)
Supplement: Supplementary file 1 — FIGURE S1 Multi‐spin‐multi‐echo (MSME) pulse sequence diagram for coronal imaging plane FIGURE S2 MREPT of phantom at 9.4T MRI. (A) Magnitude image illustrating the two electrolytes #1 and #2, and background. (B) B1 phase map after applying echo‐combination algorithm, (C) the reconstructed conductivity image. The (D) shows the profiles of B1 phase (left) and magnitude (right), along the red and black lines marked on cross‐sectional regions at the top‐right corner of the four graphs FIGURE S3 Conductivity spectra of the two electrolytes and the background agarose gel used in our phantom experiments from 10 Hz to 3 MHz. The conductivity values form the MREPT experiment also displayed at 400 MHz [file MRM-86-382-s001.docx]

**Supporting information**

**A1. Pulse Sequence Diagram**

**
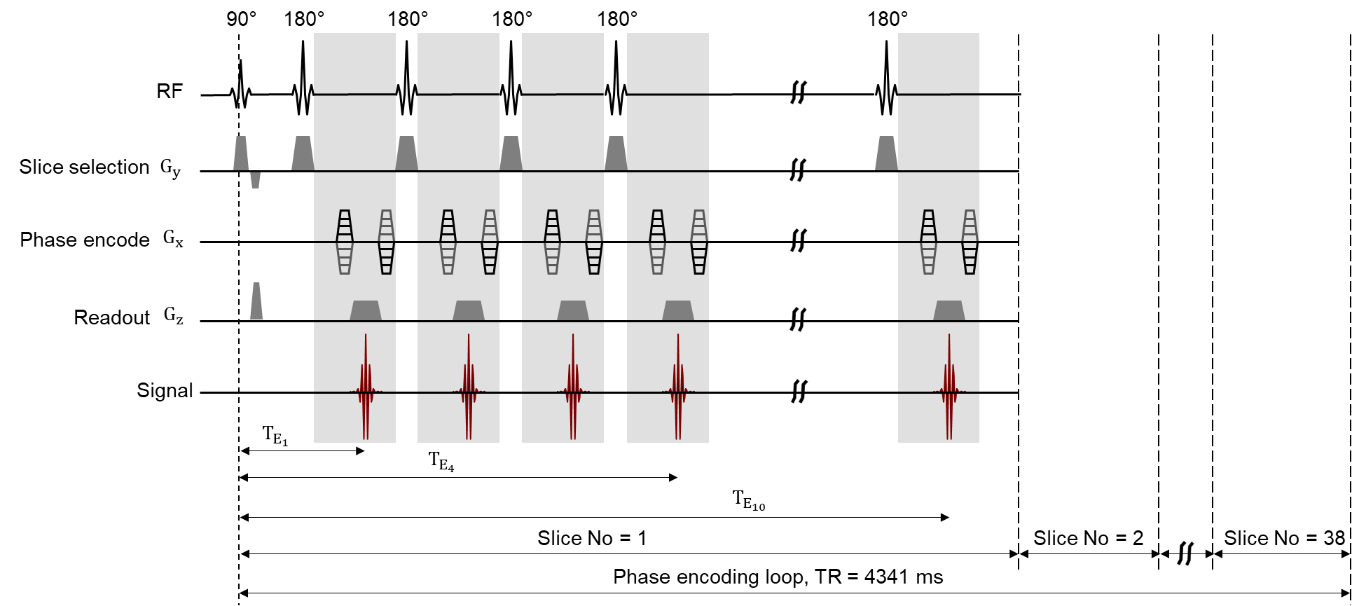
**

**Figure S1.** Multi-spin-multi-echo (MSME) pulse sequence diagram for coronal imaging plane

The MSME sequence used in this work is shown in Fig. S1 for coronal imaging plane with TR = 4341 ms. In our MSME sequence implementation, a 2D slice was selected and a train of equally spaced 180° refocusing RF pulses were played after the 90° excitation RF pulse, generating a series of spin echoes for the selected slice. The phase encoding loop was the outermost loop during data acquisition. The slice loop was inside the phase encoding loop where 10 echoes were acquired consecutively for each slice. The phase encoding gradients were balanced for each echo and remained constant during one TR. This is in-contrary to the fast spin-echo (FSE) pulse sequence used in some earlier studies where phase encoding gradients change from one echo to another acquiring multiple k-space lines within the echo loop thereby creating a T2 contrast in the acquired FSE image. Since MSME acquires all the slices and all the echoes for the slice within same TR, the resulting slice number is limited by the echo-spacing and the number of echoes.

**A2. Coil-combine method**

A birdcage transmit coil and 4-channel phase array surface receive coil was used in this work to measure the B1 map. Let us denote the $\mathbf{B}_{1}$ field in terms of its magnitude and phase in transverse plane as${: B}_{1}=\left| B_{1} \right|e^{i\phi_{1}}$. Similarly, let us denote the positive rotating component of the $\mathbf{B}_{1}$ field produced by the transmit birdcage coil as $B_{1}^{+}=\left| B_{1}^{+} \right|e^{i\phi_{1}^{+}}$ and the negative rotating component of the $\mathbf{B}_{1}$ field from the *n*th receive coil as$B_{1,n}^{-}=\left| B_{1,n}^{-} \right|e^{i\phi_{1,n}^{-}}$.

For a multi-echo pulse sequence, the complex MR image $S_{n}^{k}$ from the *n*th receive coil and *k*th echo can be expressed as (49):

$S_{n}^{k}=V_{1}M_{0}^{k}B_{1,n}^{-}e^{i\phi_{1}^{+,k}}sin{(V}_{2}\alpha\left| B_{1}^{+} \right|)=V_{1}M_{0}^{k}\left| B_{1,n}^{-} \right|e^{i\left( \phi_{1}^{+,k}+\phi_{1,n}^{-,k} \right)}sin{(V}_{2}\alpha\left| B_{1}^{+} \right|)$ (A1)

where, $M_{0}^{k}$ is the MR magnitude image containing tissue relaxation and proton density effect, *V*_1_ and *V*_2_ are system-dependent constants, and *α* is the nominal flip angle of the RF excitation. Note that $\phi_{1}^{+,k}+\phi_{1,n}^{-,k}$ is the transceive phase maps at the *nth* coil. The complex MR images from all receive coils ($S_{n}^{k})$ are combined using two-step generic referenceless phase combination (GRPC) method (30). The first step in this method, called zeroth-order correction of preliminary coil rephasing (31), is to remove the average phase of receive coils, and the second step is to use this rephased complex data in method proposed by Parker et al (52). The average phase ($\psi^{k}$) of *N_c_* receive channels for the *k*th echo is given by:

$\psi^{k}=\sum_{n=1}^{N_{c}} \left[ \frac{\phi_{n}^{k}}{\sum_{\left( x,y \right)} \left\{ \mathrm{Re}\left( \left| S_{n}^{k}\left( x,y \right) \right|e^{i\phi_{n}^{k}\left( x,y \right)} \right) \right\}} \right]$ (A2)

The zeroth-order phase approximation of $\phi_{n}^{k}$ (represented as$\Phi_{n}^{k}$), which is removing of the average phase $\psi^{k}$ from$\phi_{n}^{k}$, gives us$: \Phi_{n}^{k}=\phi_{n}^{k}-\psi^{k}$. After this, Gaussian low pass filter *Gaussian*(.) is applied on $\Phi_{n}^{k}$ and this filtered phase is subtracted again from $\phi_{n}^{k}$ as${: \theta}_{n}^{k}=\phi_{n}^{k}-Gaussian(\Phi_{n}^{k})$. The final processed phase $\theta_{n}^{k}$ is then combined by taking a complex summation over all the *N_c_* channels to get a combined signal from the multiple receive coils:

$\tilde{S}^{k}= \sum_{n=1}^{N_{c}} \left| S_{n}^{k} \right|e^{i\theta_{n}^{k}}$ (A3)

After the channels are combined in equation A3, the *N_E_* echoes are combined to create a single image for each slice as per the method described by Kwon et al (19), which is based on finding optimal weighting factor as follows:

$w_{k}=\frac{\left| \tilde{S}^{k} \right|^{2}}{\sum_{k=1}^{N_{E}} \left| \tilde{S}^{k} \right|^{2}}$ (A4)

The transceive phase map $\phi_{tr}$ was then calculated for each slice from the echo-combined data as:

$\phi_{tr}=\sum_{k=1}^{N_{E}} w_{k}.angle(\tilde{S}^{k})$ (A5)

**A3. Phantom experiment**

To demonstrate that phase-based MREPT yields reasonable results at 9.4T, we performed a phantom experiment at 9.4T Biospec MRI scanner (Bruker Biospin, Inc. Germany) with birdcage volume coil and a phased array surface coil. A cylindrical-shaped phantom of 24 mm outer diameter (22 mm inner diameter) was developed and two falcon-tubes of 5 mm diameter were inserted inside it. The two falcon-tubes were filled with different electrolytes. The electrolyte #1 and #2 were NaCl solutions of 3 g/L and 7 g/L. The background region was agarose gel mixed with a solution of 0.5 g/L of NaCl. The conductivity of the electrolytes was also measured with impedance analyzer (SI1260A, AMTEK, UK) using four-electrode method at frequency range of 10 Hz to 3 MHz.


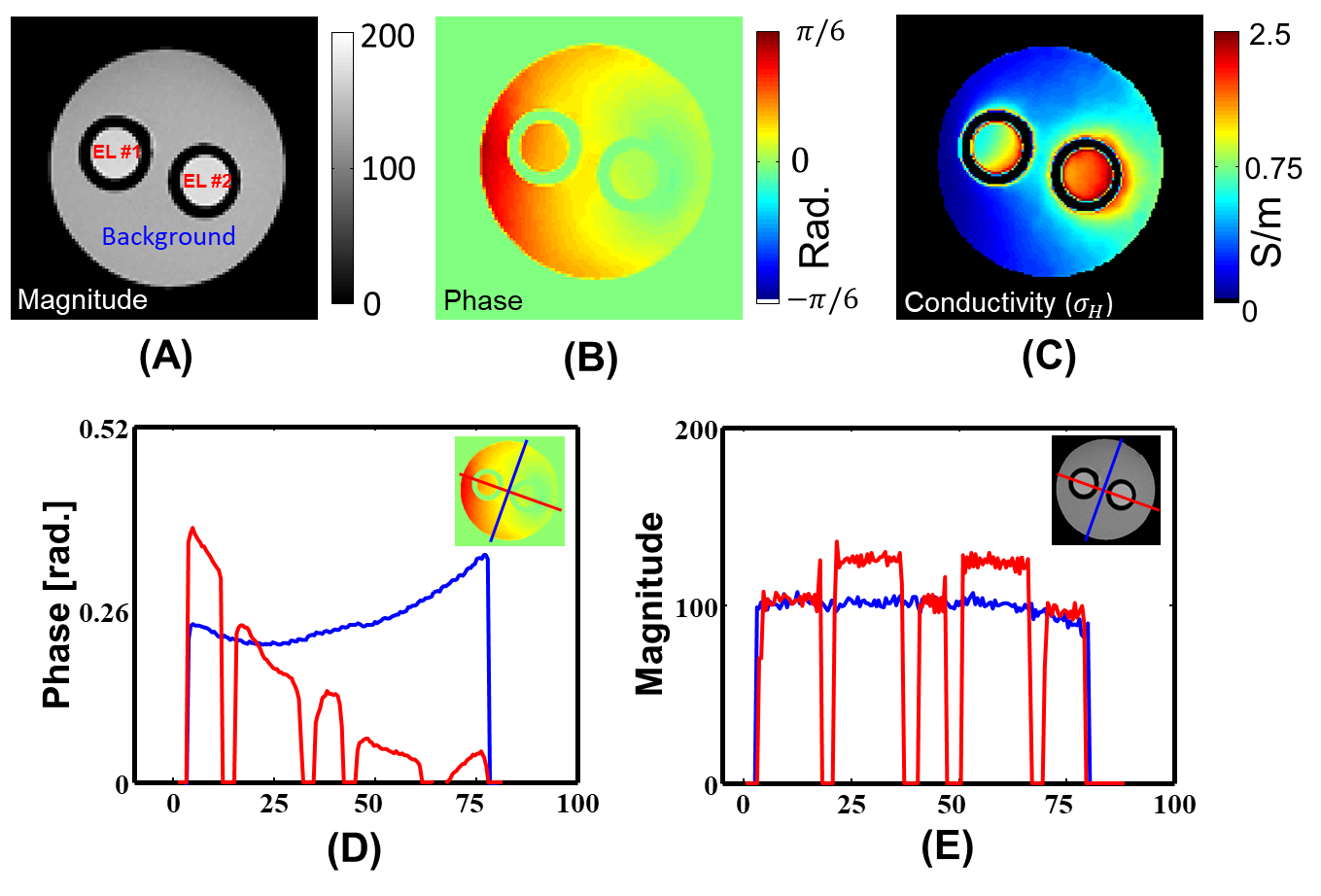


**Figure S2.** MREPT of phantom at 9.4T MRI. (A) Magnitude image illustrating the two electrolytes #1 and #2, and background. (B) B_1_ phase map after applying echo-combination algorithm, (C) the reconstructed conductivity image. The (D) and (E) shows the profiles of B_1_ phase and magnitude, along the red and blue lines marked on cross-sectional regions at the top-right corner of the two graphs.

The MR imaging parameters, phase unwrapping [32] and echo combination method [19] (section A2, equation A4 of this document) in phantom experiment was same as that used in our rat-brain experiments. The echo-combined magnitude images and the B_1_ phase map are shown in Fig S2 (A) and (B), respectively. As can be noticed from the magnitude profile plots in (E), the magnitude profiles are homogeneous which indicate that the phase-based MREPT is possible at 9.4T magnetic fields.

The conductivity images were reconstructed using the reconstruction algorithm described by Gurler et al [29] with *c­=*0.005. Figure S2 (C) shows the measured conductivity images illustrating the conductivities of electrolyte #1, #2 and background agarose gel. The reconstructed conductivity values from MREPT performed at 400 MHz and those measured using impedance analyzer at 3 MHz are also shown in Table S1 and in Fig S3. We could only measure the conductivity values from our impedance analyser at its maximum operating frequency of 3 MHz. At this frequency, the relative error of the reconstructed conductivity of electrolytes and background was in range of 23 to 36 %. However, *in-vitro* measurements at 400 MHz could be more conclusive and smaller error is anticipated.


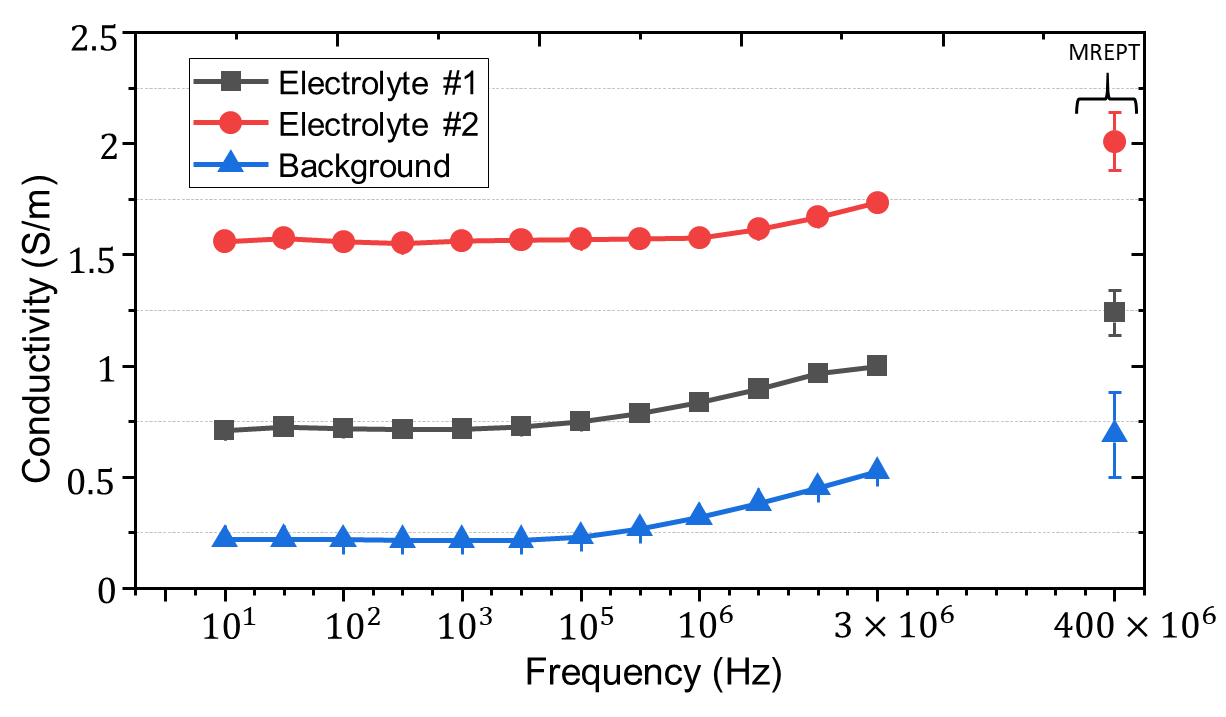


**Figure S3.** Conductivity spectra of the two electrolytes and the background agarose gel used in our phantom experiments from 10 Hz to 3 MHz. The conductivity values form the MREPT experiment also displayed at 400 MHz.

**Table S1.** Comparison between the MREPT based reconstructed conductivities of our phantom materials: agarose gel, and the two electrolytes at 400 MHz, and the impedance analyzer based measured conductivities at 3 MHz.

| **Region** | **NaCl [g/L]** | **Electrical conductivity [S/m]** | |
| --- | --- | --- | --- |
|  |  | ***In-vitro* (3 MHz)** | **MREPT (400 MHz)** |
| EL #1 | 3 | 0.79 | 1.24 $\pm$ 0.12 |
| EL #2 | 7 | 1.45 | 2.01$\pm$ 0.17 |
| Background | 0.5 + Agarose gel | 0.53 | 0.69 $\pm$ 0.19 |
